# Refractory Impella Suction Alarms in the Setting of Extracorporeal Membrane Oxygenation

**DOI:** 10.1016/j.cjco.2021.03.015

**Published:** 2021-05-02

**Authors:** Joe Aoun, Isaac Tea, Arvind Bhimaraj, Imad Hussain

**Affiliations:** a Department of Cardiovascular Medicine, Houston Methodist Debakey Heart & Vascular Center, Houston, Texas, USA; b Department of Heart Failure and Transplant Cardiology, Houston Methodist Debakey Heart & Vascular Center, Houston, Texas, USA

## Abstract

Cardiogenic shock is an uncommon but serious complication of acute myocardial infarction. Temporary mechanical circulatory support devices are being used more often in this setting, and physicians are required to be familiar with their complications. Although veno-arterial extracorporeal membrane oxygenation increases after loading, an Impella device can be inserted to unload the left ventricle and decrease its oxygen consumption. Here, we present an uncommon cause of a refractory Impella suction alarm, which was related to the migration of the venous extracorporeal membrane oxygenation cannula into the left atrium.

## Résumé

Le choc cardiogénique est une complication rare, mais grave, de l'infarctus aigu du myocarde. Des dispositifs d'assistance circulatoire mécanique temporaires sont de plus en plus utilisés dans de telles situations, et les médecins doivent bien connaître les complications possibles de ces dispositifs. Bien que l'oxygénation par membrane extracorporelle veino-artérielle augmente la postcharge, une pompe Impella peut être insérée pour décharger le ventricule gauche et diminuer sa consommation d'oxygène. Dans cet article, nous présentons une cause rare d'une alarme d'aspiration réfractaire de la pompe Impella, attribuable à la migration de la canule veineuse d'oxygénation par membrane extracorporelle dans l'oreillette gauche.

The incidence of cardiogenic shock complicating acute myocardial infarction is about 6% of cases.^1^ Despite medical and percutaneous coronary interventions (PCIs), some patients require temporary mechanical circulatory support (MCS) devices as a bridge to recovery, or to transplant or a durable left ventricular assisted device. In 2016, the Food and Drug Administration approved the Impella device (ABIOMED, Danvers, MA) for use in cases of cardiogenic shock complicating acute myocardial infarction.^2^ Physicians involved in the care of patients aided by temporary MCS devices should have knowledge of these devices and how to troubleshoot them. Here, we highlight a patient who had an Impella alarm triggered by an uncommon cause, which we found while reviewing a stepwise algorithm for troubleshooting Impella alarms in extracorporeal membrane oxygenation (ECMO) patients.

## Case

**Figure 1:**
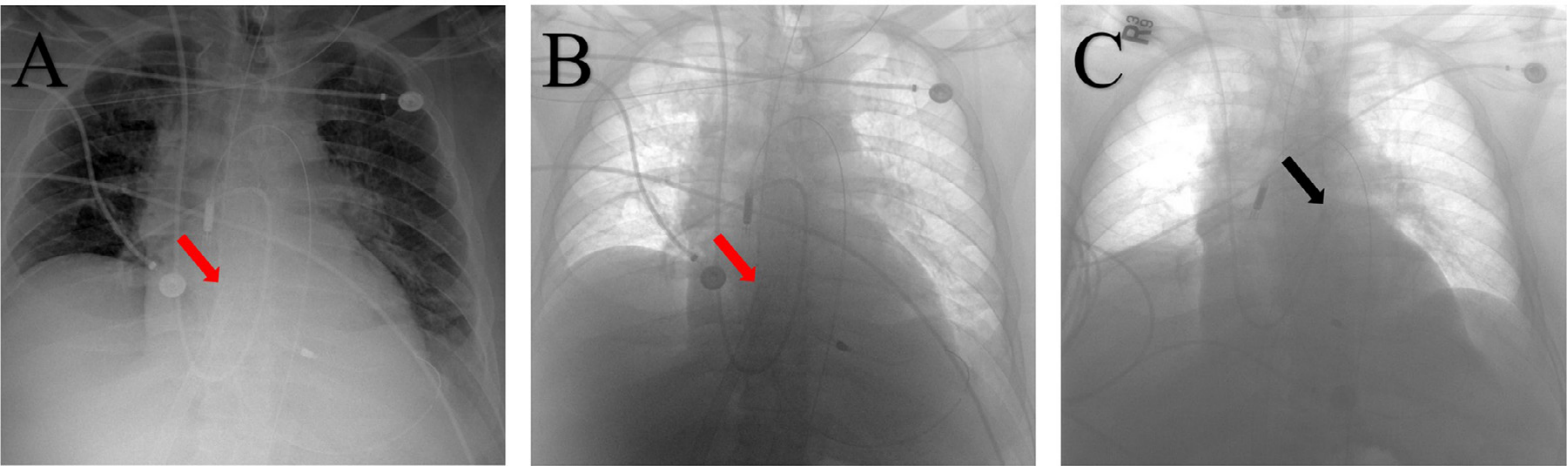
(**A, B**) Chest radiograph (normal and inverted) confirming the extracorporeal membrane oxygenation venous canula position shows slight migration from the superior vena cava–right atrium junction to the middle of the right atrium. (**C**) Inverted chest radiograph shows the migration of the venous cannula to the left atrium. **Arrows** indicate the tip of the extracorporeal membrane oxygenation venous cannula. The pulmonary artery catheter shown is deep, and the position was adjusted.

A 65-year-old man with a history of hypertension, diabetes mellitus, and smoking presented with retrosternal, nonradiating chest pain of 18-hour duration that was accompanied by shortness of breath. His physical exam was remarkable for sinus tachycardia with a heart rate of 125 beats per minute, a blood pressure of 110/68 mm Hg, and bibasilar crackles (Killip Class II). An electrocardiogram demonstrated anterior ST elevations with q waves in leads V1-V2-V3. An emergent coronary angiogram revealed a complete occlusion of the proximal left anterior descending coronary artery (culprit lesion) and a chronic total occlusion of the left circumflex coronary artery. His left ventricular end-diastolic pressure  was around 22 mm Hg. An intra-aortic balloon pump–assisted PCI was successfully performed, leading to a complete revascularization (both vessels). After the intervention, the patient became hypotensive, so the intra-aortic balloon pump was upgraded to an Impella CP. Laboratory studies revealed an elevated lactic acid level (3.3 mmol/L) and troponin level (7 ng/mL, peak > 500 ng/mL on day 1). Despite MCS, he required escalating doses of inotropes/vasopressors, so the decision was made to perform peripheral veno-arterial (VA) ECMO insertion in the catheterization laboratory, at the end of the PCI, using a Maquet 21 Fr. (PAS 2115; Maquet Medical Systems, Rastatt, Germany) left common femoral arterial and 25 Fr. (PVS 2538) left common femoral venous cannulae. Post-procedure fluoroscopy followed by chest radiograph confirmed the position of the venous cannula close to the right atrium–superior vena cava (RA-SVC) junction (Fig. 1). The Impella was left in place at P-8 (with a flow around 3.2 L/min) for left ventricular (LV) unloading. Later in the day, the Impella developed recurring suction alarms. The device was repositioned multiple times under direct echocardiographic guidance to try to stop the alarm, to no avail. His echocardiogram also showed an LV end-diastolic diameter of 5.6 cm, and an LV ejection fraction of < 20%. There was no evidence of cardiac tamponade, and fluid administration did not resolve the alarms. Furthermore, the alarms persisted despite decreasing the P-level (to P-2) of the Impella. The pulmonary artery catheter demonstrated the following hemodynamics: right atrium 15 mm Hg, pulmonary artery 24/17 mm Hg (mean: 19 mm Hg), pulmonary capillary wedge pressure 17 mm Hg, central venous pressure/pulmonary capillary wedge pressure ratio 0.88, and pulmonary artery pulsatility index 0.6. Inhaled nitric oxide was initiated with the goal of reducing right ventricular (RV) afterload; however, this did not resolve the alarms. A repeat chest radiograph noted that the venous canula of the ECMO circuit had migrated to the left atrium (Fig. 1). The venous cannula was promptly repositioned, with resolution of all Impella alarms. A transesophageal echocardiogram confirmed the presence of a patent foramen ovale (Video 1 , view video online). The Impella CP was later upgraded to an axillary Impella 5.5 device with decannulation of the ECMO on day 8. Unfortunately, the patient died a few weeks later due to significant competing risks and comorbidities that were most likely related to the late presentation of his ST-elevation myocardial infarction.

## Discussion

Impella suction alarms are commonly encountered in practice. Although the automated controller itself provides a good reference on how to deal with the whole gamut of alarms,^3^ these preset algorithms become less useful when the Impella is used in conjunction with ECMO (EC-PELLA). The Impella alarms do not take into consideration the hemodynamic effect secondary to the ECMO device or the position of its cannulae. Here, we present an uncommon cause of refractory Impella suction alarms in the setting of venous cannula migration of the ECMO circuit.

**Figure 2:**
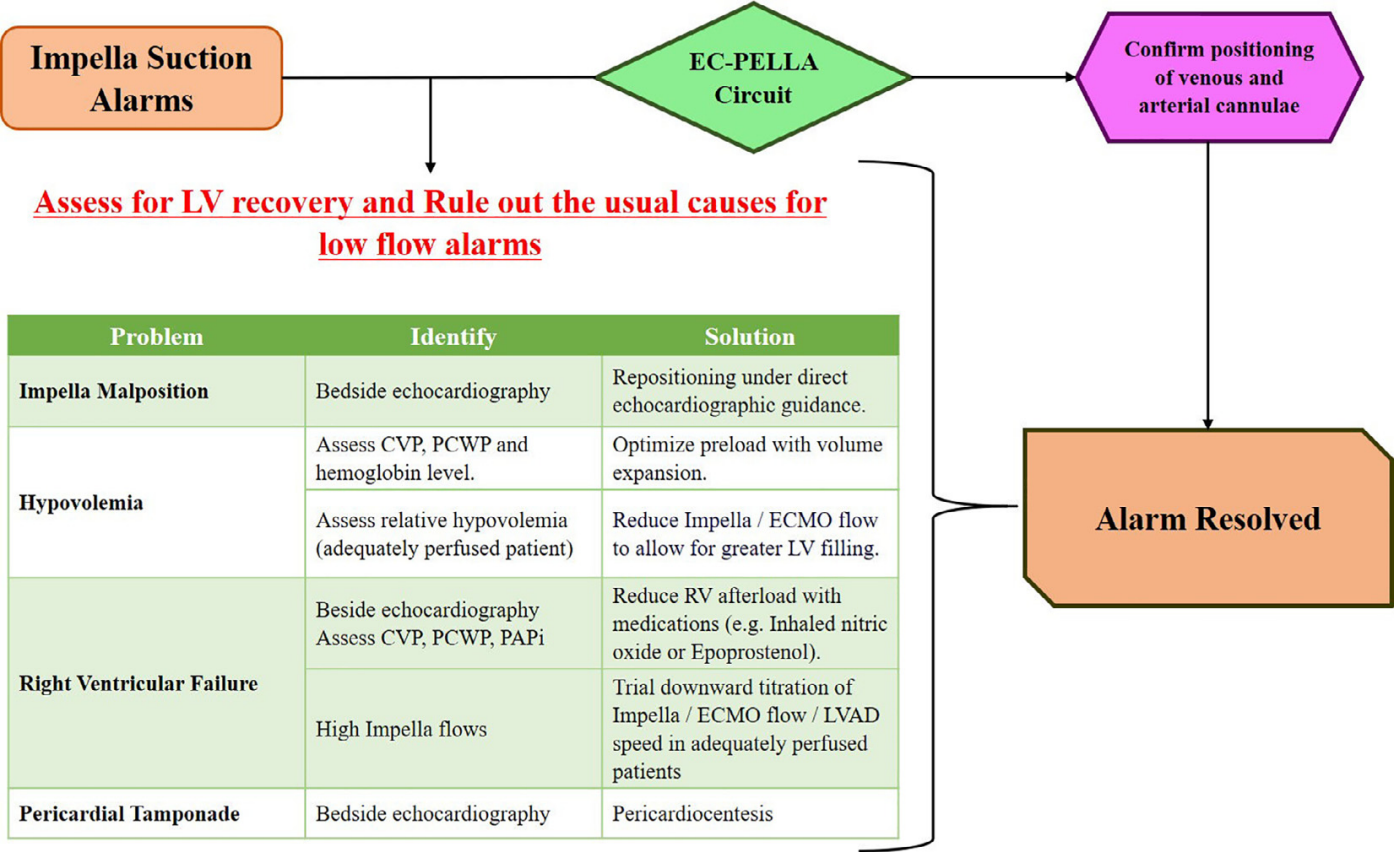
Suggested algorithm for the management of Impella suction alarms in the setting of an Impella device used in conjunction with extracorporeal membrane oxygenation (ECMO; EC-PELLA) circuit. CVP, central venous pressure; LV, left ventricular; LVAD, LV assist device; PAPi, pulmonary artery pulsatility index; PCWP, pulmonary capillary wedge pressure; RV, right ventricular.

If left alone, continuous suction alarms result in hemolysis, pump thrombosis/malfunction, and even hemodynamic instability. The most common causes of Impella suction alarms include: malposition of the Impella, hypovolemia, RV failure, and pericardial tamponade. At first, an effort should be made to identify and treat these conditions (Fig. 2). In our case, the Impella had already been confirmed to be in the appropriate position. In addition, the patient had elevated right- and left-sided filling pressures, indicating that hypovolemia was not an issue. Finally, reducing RV afterload with inhaled nitrates did not help appreciably, given that the RV function was not severely depressed. Other less common causes of suction alarms include inlet thrombus with decreased inflow (without increase in purge flow or pressure), suction of a papillary muscle/chordae into the inlet, and ischemia-related myocardial edema leading to a decreased LV cavity size.

Again, in our case, the migration of the venous cannula of the ECMO circuit through a patent foramen ovale led to a decrease in LV inflow with a subsequent smaller LV cavity, acting like a tandem heart LV support system. In other words, the EC-PELLA system had incidentally shifted from a biventricular support system to primarily LV-only support (much like an LV assist device), which can be detrimental in some patients, especially if they have advanced RV failure. Review of serial chest radiographs confirmed the diagnosis. The venous cannula was repositioned with resolution of all alarms. In conclusion, appropriate venous cannula position should be confirmed, with real-time echocardiography and/or chest radiography, at the time of veno-arterial ECMO implantation, after patient mobilization or with any change in clinical status.

## Novel Teaching Points

• In patients with ECMO, an Impella device can be inserted to unload the left ventricle; this is called an EC-PELLA system.
• Impella suction alarms are common and are caused by device malposition, hypovolemia, RV failure, pericardial tamponade, and/or LV recovery.
• Impella suction alarms in the setting of EC-PELLA can be related to cannula malposition (rarely via a patent foramen ovale).

## Funding Sources

The authors have no funding sources to declare.

## Disclosures

The authors have no conflicts of interest to disclose.

## Appendix Supplementary materials

Video 1. Transesophageal echocardiogram showing the presence of a patent foramen ovale through which the ECMO venous cannula migrated to the left atrium. Image, video 1

## Footnotes

To access the supplementary material accompanying this article, visit *CJC Open* at https://www.cjcopen.ca/ and at doi:10.1016/j.cjco.2021.03.015.
